# Does Loudness Relate to the Strength of the Sound Produced by the Source or Received by the Ears? A Review of How Focus Affects Loudness

**DOI:** 10.3389/fpsyg.2021.583690

**Published:** 2021-01-28

**Authors:** Gauthier Berthomieu, Vincent Koehl, Mathieu Paquier

**Affiliations:** Univ Brest, Lab-STICC, CNRS, UMR 6285, Brest, France

**Keywords:** loudness, auditory perception, hearing, instructions, experimental setup

## Abstract

Loudness is the magnitude of the auditory sensation that a listener experiences when exposed to a sound. Several sound attributes are reported to affect loudness, such as the sound pressure level at the listener's ears and the spectral content. In addition to these physical attributes of the stimulus, some subjective attributes also appear to affect loudness. When presented with a sound, a listener interacts with an auditory object and can focus on several aspects of the latter. Loudness appears to differ depending on how listeners apprehend this object, notably whether they focus on the sound that reaches their ears or that is produced by the source. The way listeners focus on the auditory object may depend on the stimulus itself. For instance, they might be more likely to focus on the sound emitted by the source if the latter is visible. The instructions given by the experimenters can also explicitly direct the listener's focus on the sound reaching the ears or emitted by the source. The present review aims at understanding how listeners focus on the auditory object depending on the stimuli and instructions they are provided with, and to describe how loudness depends on this focus.

## 1. Introduction

According to Florentine (2011, pp. 4–5), loudness is the perceptual strength of a sound that ranges from very soft (or quiet) to very loud. The author noted that “most definitions of loudness are somewhat vague, but most people behave in a consistent manner when judging loudness”. Loudness is known to depend on multiple factors such as the at-ear sound pressure level and the spectral content of the sound. For instance, the higher the sound pressure level is at the listener's ears, the greater its loudness generally is Stevens (1957). There is no absolute loudness value for a given sound. Rather, its assessment might vary from one listener to another, or even for the same listener during two different presentations of the sound (Algom and Marks, 1984) and depending on their mood (Siegel and Stefanucci, 2011). Loudness can also be assessed indirectly by measuring the reaction time to signal detection (Kohfeld et al., 1981), which appears to be a less subjective method but still exhibiting some variability (Schlittenlacher et al., 2014). Loudness can be estimated through models that analyze the physical properties of sounds in order to determine their typical loudness, i.e., the loudness value that would generally match the loudness values reported by a large group of human listeners (see Sivonen and Ellermeier, 2008; Moore, 2014, for examples of loudness models).

However, the link between the physical properties of a sound and the loudness experienced by the listener is not straightforward. Because loudness is a subjective experience, it depends on the way the listener interacts with the auditory object (a sound that can be assigned to a particular source following the definition of Bizley and Cohen, 2013). The environment and conditions in which the sound is presented to the listener are likely to affect the perception of this auditory object. As an example, when the source is identifiable, listeners may focus on the sound emitted by the latter (the distal stimulus) rather than on the signal reaching their ears (the proximal stimulus). This is likely to explain that loudness does not necessarily evolve in the same manner as what could be expected from variations of physical properties at the listener's ears (Zahorik and Wightman, 2001).

Listeners are able to focus on the proximal or distal stimulus when they are explicitly instructed to do so. Loudness can differ for the two cases. The assessments reported with these two distinct instructions have been described as “loudness at the ear” (Mershon et al., 1981) and “loudness at the source” (Sivonen and Ellermeier, 2011).

Loudness studies usually ask the participants to estimate the loudness of the sounds they hear without giving further specifications (see Sivonen and Ellermeier, 2006; Glasberg and Moore, 2010; Epstein and Florentine, 2012; Meunier et al., 2016). This can lead to the inter-individual variability inherent to loudness assessment (Algom and Marks, 1984; Siegel and Stefanucci, 2011; Schlittenlacher et al., 2014). By comparing the results found in the literature with different instructions and stimuli, this paper aims at understanding on what listeners focus when assessing loudness and how this focus affects their judgments. This might also help to understand differences observed in loudness assessments reported for studies that provide listeners with similar signals but with different presentation methods (Epstein and Florentine, 2009, 2012; Berthomieu et al., 2019a).

## 2. Stimulus-Driven Focus

The extent to which listeners focus on the proximal or distal stimulus while estimating loudness appears to depend on the stimulus itself. It will enable the listener to focus on its source if it contains enough information about the latter. If the stimulus does not include any information about its source, the listeners only focus on the proximal stimulus while estimating loudness. As an example, Stevens and Guirao (1962) asked their participants to estimate the loudness, softness, and apparent distance of noises and pure tones presented through headphones without any visual stimulus. Since no other information about the source was provided to the listeners, loudness, and distance estimates were solely dependent on the at-ear sound pressure level and varied inversely with each other.

### 2.1. Reverberation Cues

In reverberant environments, the direct-to-reverberant energy ratio (DRR) is an absolute distance cue (Mershon and King, 1975). This is mostly true in rooms that are sufficiently large that the reverberant energy is almost independent of sound source distance. As an example, Zahorik and Wightman (2001) measured a decrease of the diffuse reverberant energy of about 1 dB for each doubling of distance in a small auditorium with reverberation time RT_60_ of approximately 0.7 s. Since the direct energy decreases linearly with the square of distance, the difference between the direct energy and the reverberant energy is a direct cue to the source distance. Moreover, the reverberant energy is proportional to the energy delivered by the sound source and could be a direct cue to the latter. Thus, reverberant environments simultaneously provide the listener with distance and power information about the source. When listeners evaluate loudness, they might focus on the loudness of the distal stimulus by following two distinct approaches: directly focusing on the source power via the reverberant energy or combining the source distance perceived through the direct-to-reverberant energy ratio and the perceived level of the proximal stimulus. Zahorik and Wightman (2001) observed what they defined as loudness constancy (loudness remained constant despite physical changes in the stimulus) using noise bursts presented virtually at several distances from the listening point in the aforementioned environment. The stimuli were presented over headphones after being binaurally recorded in the environment and were thus not visible during the experiment. Listeners gave constant loudness estimates for sounds played at different distances by a source of constant power despite at-ear sound pressure level differences, in agreement with Altmann et al. (2013) who reported that reverberation cues are used to achieve constant loudness across distance. Zahorik and Wightman (2001) suggested the hypothesis that loudness constancy is not related to perceived distance on the basis of two arguments. Firstly, they asked the participants to verbally estimate the distance of the sound sources for which loudness constancy was observed and obtained discrepancies between the estimates and the actual distances. Nevertheless, such discrepancies could be accounted for by the distance assessment method (verbal report) which is reported to lead to systematic underestimation (Paquier et al., 2016) and to be less accurate than proprioceptive methods such as blind walking (Andre and Rogers, 2006). Secondly, loudness constancy was not observed at low source power levels, for which the reverberant field fell below the absolute threshold of hearing. However, the absence of a perceptible reverberant field might not only have removed the information about the power of the source, but also about its distance.

### 2.2. Timbral Cues

For stimuli such as speech or music, intrinsic information about the sound source can be conveyed through the sound timbre. Speech perception is not solely based on the extraction of simple physical parameters conveyed in the speech waveform (Moore, 2012). The perceived vocal effort of a speaker can give information about the source power (Rosenblum and Fowler, 1991), allowing the listeners to evaluate the strength of the emitted speech at the position of the speaker regardless of the level of the sound reaching their ears. Mohrmann (1939) asked listeners to adjust the output level of two sound sources positioned at different distances so that the two sources appeared to be equally loud. The sounds included speech, music, tones and noises, and the sources could be either visible or hidden. The results showed that the output levels set by the listeners were less dependent on the source distance for speech and music than for tones and noises. Thus, listeners focused more on the source for speech and music than for tones and noises, for which loudness estimates were more related to the strength of the sound reaching their ears. The output levels were also less dependent on the sources distances when the sources were visible. The distance cues provided by vision are reported to enhance the accuracy of distance judgements (Anderson and Zahorik, 2014) and are likely to help the participants to focus on the sound source by giving more accurate information about it, as discussed in the following subsection. Pollack (1952) and Warren (1973) asked their participants to compare the loudness of two sounds (that could be noises, pure tones, and speech) played at different levels. The results showed a weaker dependence of the loudness on the at-ear sound pressure level for speech than for noises and pure tones. Loudness comparisons for noises and pure tones were highly dependent on the level of the sounds reaching the listeners ears. The speech stimuli were always the same recording played at several levels. Thus, the at-ear sound pressure varied accordingly to the output level but the timbre was the same regardless of the output level. Since loudness estimates depended less on the at-ear sound pressure level, listeners might have taken into account the perceived invariant level of the original stimulus (whose constant strength was perceived via the vocal effort regardless of the at-ear sound pressure level) in their loudness estimates. Even though listener's focus was not explicitly driven on the distal or proximal stimulus, this focus was likely to have been more spontaneously put on the source for speech stimuli than for noises or tones.

Epstein and Florentine (2009) observed stronger loudness constancy in the binaural-to-monaural loudness ratio for speech than for pure tones, despite similar physical variations in the sound properties. Loudness estimates were gathered for pure tones and speech stimuli played to either one or both ears. Pure tones were perceived as significantly louder when presented binaurally than monaurally, in agreement with Fletcher and Munson (1933). The binaural-to-monaural loudness ratio was significantly smaller for speech stimuli. The intrinsic source information conveyed by speech could have led to the perception of an auditory object that naturally directs the focus toward the source, which strength might be acknowledged by the listeners to be independent on whether it is heard monaurally or binaurally (Culling and Dare, 2016).

### 2.3. Visual Cues

In a follow-up study using the same procedure as for their 2009 paper, Epstein and Florentine (2012) reported that binaural-to-monaural loudness ratio was significantly smaller for speech stimuli when the speaker was visible. Thus, visual cues might help the listeners to focus on the source. Rosenblum and Fowler (1991) gathered loudness estimates using graphic ratings. Videotapes of a speaker producing consonant-vowel utterances and of hand claps were presented to listeners, whose task was to adjust the position of a vertical slash mark on an horizontal line in a location that corresponded with their impression of loudness, with increasing loudness corresponding to increasing distance from the left end of the line. The auditory and visual stimuli were produced at four degrees of efforts, and could be presented with or without a discrepancy between the auditory and visual efforts. The loudness estimates were affected significantly by the effort apparent in the visual stimuli. Thus, listeners focused on the source thanks to non-auditory information while estimating loudness. Shigenaga (1965) asked listeners to adjust the output level of sources positioned at different distances so that they appeared to play sounds as loud as for a reference source positioned at a fixed distance. The sources were visible, in an environment with low reverberant energy (the experiment took place on a roof, with participants sitting on elevated chairs so that their heads were 3.3 m above the roof surface). The output powers of the sources adjusted this way were similar despite the at-ear sound pressure variations induced by the distance differences, showing loudness constancy with source distance.

Namba et al. (1997) gathered loudness ratings for car interior sounds presented with different videos filmed through a front car window. The videos showed different ways of driving (e.g., busy roads with a high amount of traffic or clear mountain areas), giving different information about how the car was running. The loudness ratings were highly dependent on the videos that were used. Videos of comfortable driving led to lower loudness. According to Menzel et al. (2008), the color of a car also has a small influence on its loudness for German listeners as the presentation of a red car produced higher loudness ratings compared to other colors. Suzuki et al. (2000) asked listeners to evaluate broadband noises that were difficult to identify with no visual information (such as the roaring of a waterfall). The noises were presented alone or with visual or verbal information about their source. The evaluations were made with pairs of verbal attributes. Based on the use of adjectives relative to loudness, such as powerful, loud, and noisy, the authors suggested that loudness was affected by the visual and verbal information provided about the sources. Berthomieu et al. (2019a) evaluated the directional loudness (i.e., the variation of loudness with the direction of the source) of narrow-band noise bursts in a sound-attenuated room. Loudness assessments were made using two experimental setups, one where the sounds were presented by visible loudspeakers, and one where the sounds were binaurally recorded and played through headphones, with no visual information about the sources. The loudness varied more with the source direction when the sounds were played through headphones (with no visual information about the sources) than when the sounds were played by the visible sources positioned around the listener. When no visual information about the source was available, estimates might have been made only with regard to the proximal stimulus. When information about the source was available through vision, listeners could have focused on the source and evaluated the distal stimulus.

## 3. Instruction-Driven Focus

In some studies, the experimenters chose to explicitly drive the focus of the participants on the proximal or distal stimulus. These studies are rather sparse, but show a strong influence of the instructions on loudness.

The instructions given by Mohrmann (1939) that led to the aforementioned data were to adjust the output levels of the sources so that “the two sources—or else the two impacts—appeared to be equally loud,” either based on “attitude toward loudness of sound emitted at the source” and “attitude toward loudness of impact at the ear” (as translated by Brunswik, 1956, p. 71). The adjustments made with the “attitude toward loudness of impact at the ear” were highly dependent on the sound source distance (and thus on the level of the proximal stimulus), which was not the case when the attitude was “toward loudness of sound emitted at the source.”

The aforementioned data obtained by Zahorik and Wightman (2001, p. 83) were collected “using a free-modulus magnitude estimation procedure in which listeners were carefully instructed to make their judgments based on the sound source power.” As described above, the loudness estimates gathered in this way showed that loudness did not vary with source distance. Listeners were able to take into account the power of the source, which was the same at every distance. When the reverberant energy fell under the absolute threshold of hearing, listeners could not focus on the source anymore and loudness estimates varied with the source distance.

Honda et al. (2019) asked participants to match the loudness of a target sound (2-s tones produced by an actual musical instrument performance at different distances from the listeners) by using two adjustment methods. They were either instructed to play a musical instrument (a melodica) as loudly as the target (“sound production”) or to adjust the sound emitted by a loudspeaker so that it had the same loudness as the target (“sound level adjustment”). The loudness obtained through the sound production method depended less on the source distance than the sound level adjustment method, especially when visual cues about musical performance were available. This suggests that the sound production method combined with visual cues enabled the participants to focus on the source.

Rosenblum and Fowler (1991) gathered the aforementioned loudness estimates using graphic ratings (where listeners adjusted the position of a vertical slash mark on an horizontal line as described above). The sounds were consonant-vowel utterances and hand claps produced with different degrees of effort. They instructed their listeners to base their loudness judgments only on what they heard, despite the sound sources (the speaker or the person clapping their hands) being visible. Listeners were this way asked to focus on the sound only, but with no particular focus on the proximal or distal stimulus. Visual effort still affected loudness estimates, showing that listeners interacted with the audiovisual object despite being asked to focus on the sound only.

Listeners are nevertheless able to evaluate the loudness of the proximal stimulus, when instructed to do so, by ignoring the available information about the source. Berthomieu et al. (2019b) asked listeners to estimate the distance and loudness of sounds played at distances ranging from 1 to 16 m by both visible and hidden sound sources in both anechoic and reverberant environments. Listeners were explicitly instructed to report the apparent loudness of the sound reaching their ears using an absolute magnitude estimation. The perceived distance was estimated in meters. Loudness estimates depended on distance and thus on the at-ear sound pressure level. Moreover, no difference was observed between loudness estimates for visible and hidden sources (in either the anechoic or reverberant environment). Distance estimates were closer to the physical sound source distances for visible sources than for hidden sources. Thus, although visual cues provided the listeners with additional information about the sources that improved their distance estimates, loudness estimates were unchanged. Listeners might then have focused on the proximal stimulus whether or not the stimuli provided information about the source.

## 4. Discussion

Since the definition of loudness itself is somewhat vague (Florentine, 2011) as the perceptual strength of the “sound,” it may vary from one listener to another or from one experimental setup to another. Some experimenters have assumed to be more specific by focusing the listeners toward the sound emitted by the source (the distal stimulus) or reaching the ears (the proximal stimulus). This focus can be obtained from the stimulus through the information it conveys about its source (reverberation cues, visual cues, timbral cues) and from the related instructions. However, listeners might not be able to follow such instructions. As an example, even though the listeners are instructed to evaluate the loudness of the proximal stimulus, the judgments may still be influenced by available information about the source. Rosenblum and Fowler (1991) reported that listeners failed to focus on the proximal stimulus when provided with visual cues to the source in presentation conditions that could exhibit discrepancies between the visual and auditory stimuli.

Loudness experiments usually do not require the listeners to specifically focus on the proximal or distal stimulus. Rather, instructions are often free (e.g., assess the perceptual strength of the sound), for example in studies of directional loudness. Such studies show loudness variations according to the direction from which the sounds reach the listener (Sivonen and Ellermeier, 2006; Kopčo and Shinn-Cunningham, 2011; Koehl and Paquier, 2015; Meunier et al., 2016). Most of these variations are accounted for by physical binaural parameters such as the interaural time or level differences. However, significant individual differences were observed by Sivonen and Ellermeier (2006) and Meunier et al. (2016) and were hypothesized to be accounted for by different degrees of loudness constancy. Provided that the sources were visible in these experiments, some listeners would have assessed the (constant) loudness of the distal stimuli while others judged the proximal stimuli. This is supported by recent results (Berthomieu et al., 2020) that reported loudness constancy when explicitly asking the listeners to assess the loudness of the distal stimulus, but not when explicitly asking the listeners to assess the loudness of the proximal stimulus.

An example that highlights such a difference is a listener who evaluates the loudness of a siren played at different distances. If the listener is asked to focus on the sound reaching the ears, the estimates might strongly depend on the alarm distance since the latter induces at-ear sound pressure level variations of the stimulus on which they focus. If the listener is asked to focus on the source, the estimates might be constant with the alarm distance (and with the at-ear sound pressure level) since its timbre gives the listener an impression of the source power, which does not depend on the source distance. If the instructions do not ask to focus on the proximal or distal stimulus, listeners might focus on either while estimating loudness. If the siren is distant, the at-ear sound pressure level might be weak and the listener might assign a low loudness value to this stimulus. On the other hand, when the sound is recognized as a siren alarm—which is known by experience to be intense—the listener might assign a high loudness as this intensity is part of the source identity (Traer et al., 2020).

The way listeners focus on the sound when asked to evaluate loudness with no further specification is difficult to evaluate. Instructions might also be differently understood by various listener panels because of cultural differences. As an example, the loudness of passing-by train noises obtained with a magnitude estimation protocol appeared to be influenced by the train color for German and Japanese listeners (Patsouras et al., 2002; Rader et al., 2004), but not for French ones (Parizet and Koehl, 2011).

## 5. Conclusion

The results reviewed show that loudness assessments depend on what the listener focuses on when estimating loudness. According to the instructions they are given and to the quantity and quality of information provided about the sound source, loudness might relate to the strength of the sound emitted by the source (the distal stimulus) or received by the ears (the proximal stimulus). These two percepts do not depend on the physical attributes of the sound in the same way, and the listener's focus might vary from one listener to another in a same experiment. These observations could thus account for results in the literature according to which some parameters (sound pressure level, source position, monaural vs. binaural listening...) have a weaker effect on the loudness of sounds whose source is identifiable by the listener and where individual differences are observed.

## Author Contributions

This bibliographic review was part of GB's Ph.D. thesis focusing on the influence on source position on loudness. VK and MP supervised and directed GB's work during his Ph.D. thesis. All authors contributed to the article and approved the submitted version.

## Conflict of Interest

The authors declare that the research was conducted in the absence of any commercial or financial relationships that could be construed as a potential conflict of interest.
